# Amelioration of galactosamine-induced nephrotoxicity by a protein isolated from the leaves of the herb, *Cajanus indicus *L

**DOI:** 10.1186/1472-6882-7-11

**Published:** 2007-04-25

**Authors:** Mahua Sinha, Prasenjit Manna, Parames C Sil

**Affiliations:** 1 Department of Chemistry, Bose Institute, 93/1, Acharya Prafulla Chandra Road, Kolkata-700009, India

## Abstract

**Background:**

Galactosamine (GalN), an established experimental toxin, mainly causes liver injury via the generation of free radicals and depletion of UTP nucleotides. Renal failure is often associated with end stage liver damage. GalN intoxication also induces renal dysfunction in connection with hepatic disorders. Present study was designed to find out the effect of a protein isolated from the leaves of the herb *Cajanus indicus *against GalN induced renal damage.

**Methods:**

Both preventive as well as curative effect of the protein was investigated in the study. GalN was administered intraperitoneally at a dose of 800 mg/kg body weight for 3 days pre and post to protein treatment at an intraperitoneal dose of 2 mg/kg body weight for 4 days. The activities of antioxidant enzymes, superoxide dismutase (SOD), catalase (CAT), glutathione reductase (GR) and glutathione-S-transferase (GST), levels of cellular metabolites, reduced glutathione (GSH), total thiols, oxidized glutathione (GSSG) and lipid peroxidation end products were determined to estimate the status of the antioxidative defense system. In addition, serum creatinine and urea nitrogen (UN) levels were also measured as a marker of nephrotoxicity.

**Results:**

Results showed that GalN treatment significantly increased the serum creatinine and UN levels compared to the normal group of mice. The extent of lipid peroxidation and the level of GSSG were also enhanced by the GalN intoxication whereas the activities of antioxidant enzymes SOD, CAT, GR and GST as well as the levels of total thiols and GSH were decreased in the kidney tissue homogenates. Protein treatment both prior and post to the toxin administration successfully altered the effects in the experimental mice.

**Conclusion:**

Our study revealed that GalN caused a severe oxidative insult in the kidney. Protein treatment both pre and post to the GalN intoxication could protect the kidney tissue against GalN induced oxidative stress. As GalN induced severe hepatotoxicity followed by renal failure, the protective role of the protein against GalN induced renal damages is likely to be an indirect effect. Since the protein possess hepatoprotective activity, it may first ameliorate GalN-induced liver damage and consequently the renal disorders are reduced. To the best of our knowledge, this is probably the first report describing GalN-induced oxidative stress in renal damages and the protective role of a plant protein molecule against it.

## Background

Galactosamine (GalN) is a well known toxin and has been found to possess various adverse effects in liver. Its toxic effect is associated with the depletion of UTP nucleotides followed by the formation of UDP hexosamines [1] and leads to the inhibition of transcription and consequently the translation processes [2]. The development of acute liver failure due to the administration of GalN is well documented [3-5]. Javle et al reported that GalN-induced liver injury is associated with the development of renal failure [6]. Recently, the term hepatorenal syndrome (HRS) has been introduced to define the development of renal failure in the absence of clinical, anatomical or pathological causes of that failure. Classically, HRS is associated with the end-stage liver cirrhosis and it has been observed that renal failure occurs with this liver disease in about 50% of patients [7]. A very recent report of Anand et al revealed that renal failure appeared with acute liver damage where the histological status of the kidney was normal but the renal blood flow was decreased [8]. Although oxidative stress has been reported as one of the major causes in GalN induced liver damages [9-11], it is not, however, clear whether GalN-induced renal disorders occur via the generation of reactive oxygen species followed by oxidative stress in the kidney or not.

Herbal medicines have been used traditionally world wide for the prevention and treatment of various diseases. Earlier studies reported that medicinal herbs play a protective role against GalN induced liver damage [12-14]. In India medicinal plants are widely used in the ayurvedic medicines for the treatment of various health problems. Some of the important medicinal plants are *Silybum marianum *[15,16]; *Picrorhiza kurroa *[17]; *Curcuma longa *[18]; *Phyllanthus niruri *[19-23]; *Terminalia arjuna *[24-26]; *Andrographis paniculata *[27] etc. The herb *Cajanus indicus*, an important medicinal plant, has been widely used in folklore medicine in India and many other countries for the treatment of various hepatic disorders [28,29]. In our laboratory a 43 kD protein has been isolated and purified to homogeneity from the leaves of this herb [30] and believed to be an active principle responsible for its beneficial action. It has been found that that the protein possesses a protective role against various toxins (such as carbon tetrachloride, chloroform, thioaectamide etc.) induced hepatic disorders [31-34]. The free radical scavenging activity of this protein was determined from its DPPH radical quenching ability [31]. The present study was conducted to find out whether GalN caused renal damage by inducing oxidative stress in the kidney and whether the protein of our interest could ameliorate that effect. Experiments were designed accordingly to evaluate both the preventive as well as curative role of this protein against GalN induced renal toxicity in vivo. The extent of renal damage caused by GalN intoxication and the protective effect of the protein were evaluated by measuring the i) level of serum creatinine and urea nitrogen (UN) ii) activities of the intracellular enzymes namely, superoxide dismutase (SOD), catalase (CAT), glutathione reductase (GR) and glutathione-S-transferase (GST) iii) levels of cellular metabolites like reduced glutathione (GSH), total thiols, oxidized glutathione (GSSG) and iv) extent of lipid peroxidation in the kidney tissue homogenates of all the experimental animals.

## Methods

### Plant

*Cajanus indicus *is a shrub belonging to the family Leguminosae and subfamily papilionacea. Fresh young leaves were collected from Bose Institute experimental farm.

### Animals

Swiss albino mice (male, body weight 20 ± 2 g) were used for the experiments. The animals were acclimatized under standard laboratory conditions for a fortnight before starting the experiments. They were provided with standard diet and water ad libitum. They were maintained under standard conditions of temperature (30°C) and humidity (50%) with an alternating 12 hours light/dark cycles. All the experimental studies were conducted in conformity with the guidance for care and standard experimental animals study ethical protocols. The animals were divided into several groups, each group having six mice.

### Chemicals

Bovine serum albumin (BSA) and Bradford reagent were purchased from Sigma-Aldrich Chemical Company, (St. Louis, MO) USA. Kit for determination of creatinine and UN were purchased from Span Diagnostic Ltd. Ammonium sulphate [(NH_4_)_2_SO_4_], 1- chloro-2,4-dinitrobenzene (CDNB), D(+)-galactosamine hydrochloride (GalN), 5,5'-dithiobis(2-nitrobenzoic acid) [DTNB, (Ellman's reagent)], ethylene diamine tetraacetic acid (EDTA), N-ethylmaleimide (NEM), nicotinamide adenine dinucleotide reduced (NADH), nitro blue tetrazolium (NBT), oxidized glutathione (GSSG), phenazine methosulphate (PMT), potassium dihydrogen phosphate (KH_2_PO_4_), reduced glutathione (GSH), sodium pyrophosphate, trichloro acetic acid (TCA), thiobarbituric acid (TBA), vitamin E were bought from Sisco Research Laboratory, India.

### Preparation of the homogeneous protein from the leaves of *Cajanus indicus*

The protein was purified from the leaves of *Cajanus indicus *according to the method described by Sarkar et al [30]. The leaves were homogenized in 20 mM tris-HCl buffer, pH 7.4 and the supernatant was brought to 60% (NH_4_)_2_SO_4 _saturation. The pellet was reconstituted and dialyzed in tris-HCl buffer, passed through DEAE Sephadex column and eluted in linear gradient of 0–1 M NaCl in tris buffer. The active fraction eluted at 0.2 M NaCl was concentrated and applied on a Sephadex G-50 column. The bioactive fraction obtained was subjected to a C_18 _hydrophobic column for reverse phase column chromatography. The homogeneity of preparation was determined by SDS-polyacrylamide gel electrophoresis (SDS-PAGE).

### Protein estimation

The protein concentration of the experimental samples was measured according to the method of Bradford [35] using crystalline BSA as standard.

### Pre treatment with the protein

The pre-treatment group was divided into five sub-groups each consisted of six mice. The first group, which received only the vehicle, served as normal control. The second group, served as toxin control, in which GalN was administered intraperitoneally at a dose of 800 mg/kg body weight once daily and the treatment was carried out for 3 days. The third group of mice was pre-treated with the protein by intraperitoneal injection at a dose of 2 mg/kg body weight once daily and the treatment was carried out for 4 days followed by the toxin treatment for another 3 days. The animals were then sacrificed under anesthesia and kidneys were collected. A well-known antioxidant agent vitamin E, (at a dose of 200 mg/kg body weight) and a non-relevant protein, BSA (at a dose of 2 mg/kg body weight) were used as the positive and negative controls respectively for the study.

### Post-treatment with the protein

The post-treatment group was divided into five sub-groups each consisted of six mice. The first group, served as normal control, received only the vehicle. The second group received GalN at a dose of 800 mg/kg body weight intraperitoneally (once daily) for 3 days and served as the toxin control. The third group of mice was intoxicated with GalN (800 mg/kg body weight, once daily) for 3 days followed by intraperitoneal administration of the protein at a dose of 2 mg/kg body weight once daily for 4 days. The fourth group received GalN (800 mg/kg body weight, once daily) for 3 days and then kept for another 4 days without any treatment to observe whether there is any natural recovery or not. A positive control group was kept in which mice were treated with 200 mg/kg body weight vitamin E for 4 days after GalN intoxication. Mice were then sacrificed under anesthesia and kidneys were collected.

### Preparation of kidney homogenates

The kidney tissues were homogenized using glass homogenizer in 100 mM potassium phosphate buffer containing 1 mM EDTA, pH 7.4 and centrifuged at 12,000 g for 30 minutes at 4°C. The supernatant was collected and used for following experiments.

### Assessment of marker of nephrotoxicity

#### Estimation of creatinine level

Blood samples collected from puncturing mice hearts were kept overnight at 4°C to clot and then centrifuged at 3,000 g for 10 minutes. Creatinine level was measured from serum samples according to the method of Bonses and Taussky [36].

#### Estimation of UN level

Estimation of UN level has been carried following the method of Crocker [37]. Like creatine measurement, blood samples were collected from puncturing mice hearts and kept overnight at 4°C to clot. Sera from the samples were collected by centrifuging the blood samples at 3,000 g for 10 minutes. UN level in all the experimental samples were measured by using a kit from Span Diagnostics Limited, India.

### Estimation of lipid peroxidation end products

The extent of lipid peroxidation in terms of thiobarbituric acid reactive substances (TBARS) formation was measured according to the method of Esterbauer and Cheeseman [38] using the extinction co-efficient of MDA, which is 1.56 × 10^5 ^M^-1^cm^-1^.

### Assay of antioxidant enzymes

#### SOD assay

The activity of SOD was measured following the method originally developed by Nishikimi [39] and then modified by Kakkar [40]. One unit of SOD activity is defined, as the enzyme concentration required inhibiting chromogen production by 50% in one minute under the assay condition.

#### CAT assay

The enzyme CAT converts H_2_O_2 _into water. The CAT activity was measured by the method of Bonaventura [41]. One unit of CAT activity is defined as the amount of enzyme, which reduces 1 μmol of H_2_O_2 _per minute.

#### GST assay

GST catalyzes the conjugation reaction with glutathione in the first step of mercapturic acid synthesis. The activity of GST was measured by the method of Habig and Jakoby [42]. One unit of GST activity is 1 μmol product formation per minute.

#### Assay of GR

GR activity was measured according to the method of Smith [43]. The enzyme activity was calculated using molar extinction coefficient of 13,600 M^-1^cm^-1^. One unit of enzyme activity is defined as the amount of enzyme, which catalyzes the oxidation of 1μmol NADPH per minute.

### Assay of cellular metabolites from kidney homogenates

#### GSH assay

GSH level was measured according to the method of Ellman [44]. A standard curve was drawn using different known concentrations of GSH solution. With the help of this standard curve, GSH contents were calculated.

#### GSSG assay

GSSG level was measured according to the method of Hissin and Hilf [45]. The absorbance of the sample was measured at 412 nm.

#### Assay of total thiols

The total thiols (total sulfhydryl groups) content was measured according to the method of Sedlak and Lindsay [46] with some modifications. The content of total thiols was calculated using molar extinction coefficient of 13,600 M^-1^cm^-1^.

### Statistical analysis

All the values are represented as mean ± S.D. (n = 6). The statistical differences among different groups were analised by Student's *t*-test. P values of 0.05 or less were considered significant.

## Results

### Effect on nephrotoxicity markers

#### Creatinine levels

**Figure 1:**
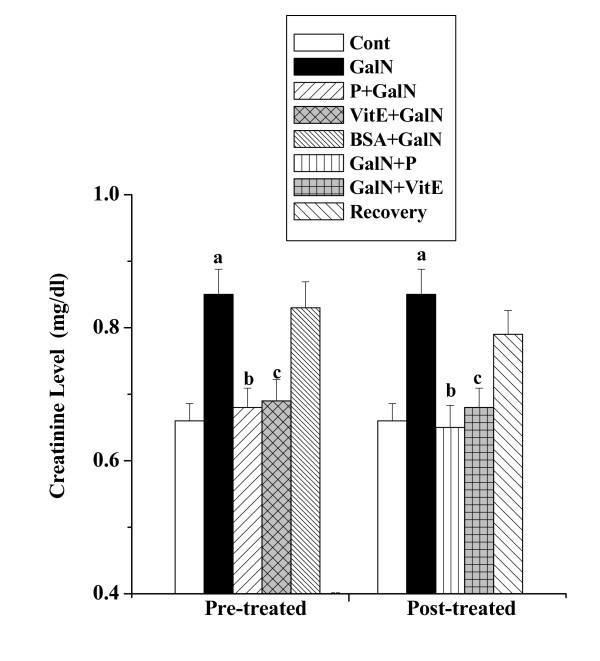
Effect of the protein on creatinine level in GalN induced kidney damage in mice. Left and right panel show preventive and curative effect of the protein respectively. Cont: creatinine level in normal mice, GalN: creatinine level in only GalN treated mice, Protein + GalN: creatinine level in which the protein was given prior to GalN administration, VitE + GalN: creatinine level in which vitamin E was administered prior to GalN treatment, BSA + GalN: creatinine level in which BSA was given before GalN administration. GalN + Protein: creatinine level in which the protein was given after GalN administration, GalN + Vit E: creatinine level in which vitamin E was administered post to GalN treatment and Recovery: creatinine level in mice which were treated with GalN for 3 days and kept next 4 days without any treatment. Each column represents mean ± SD, n = 6. "a" indicates the significant difference between the vehicle control and toxin treated groups, "b" indicates the significant difference between the toxin treated and protein treated groups and "c" indicates the significant difference between the toxin treated and vitamin E treated groups. (P^a ^< 0.01, P^b ^< 0.01, P^c ^< 0.01).

The creatinine levels in serum obtained from the normal control mice, toxin control mice, mice treated separately with protein, vitamin E and BSA followed by GalN intoxication, mice treated separately with protein, vitamin E after GalN intoxication and mice kept for normal recovery after GalN administration are shown in figure 1. The creatinine level was elevated in toxin control (0.85 ± 0.038 mg/dl) compared to the normal control (0.66 ± 0.026 mg/dl), whereas protein pre-treatment reduced the level (0.68 ± 0.029 mg/dl). There was reduction of creatinine level (0.65 ± 0.033 mg/dl) in protein post- treated group of mice. The normal recovery after toxin administration was very slow (0.79 ± 0.036 mg/dl).

#### UN levels

**Figure 2:**
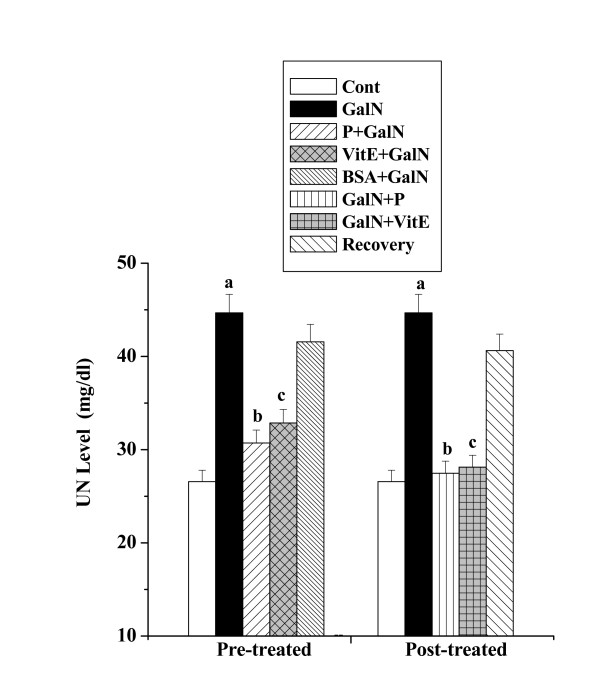
Effect of the protein on UN level in GalN induced kidney damage in mice. Left and right panel show preventive and curative effect of the protein respectively. Cont: UN level in normal mice, GalN: UN level in only GalN treated mice, Protein + GalN: UN level in which the protein was given prior to GalN administration, VitE + GalN: UN level in which vitamin E was administered prior to GalN treatment, BSA + GalN: UN level in which BSA was given before GalN administration. GalN + Protein: UN level in which the protein was given after GalN administration, GalN + Vit E: UN level in which vitamin E was administered post to GalN treatment and Recovery: UN level in mice which were treated with GalN for 3 days and kept next 4 days without any treatment. Each column represents mean ± SD, n = 6. "a" indicates the significant difference between the vehicle control and toxin treated groups, "b" indicates the significant difference between the toxin treated and protein treated groups and "c" indicates the significant difference between the toxin treated and vitamin E treated groups. (P^a ^< 0.01, P^b ^< 0.01, P^c ^< 0.01).

Figure 2 represents UN level in serum of all the experimental animals. In toxin control an increase in UN level (44.68 ± 1.98 mg/dl) has been observed compared to the normal control (26.57 ± 1.22 mg/dl), whereas protein pre-treatment attenuated the level (30.71 ± 1.39 mg/dl). Protein post treatment also reduced the UN level (27.47 ± 1.29 mg/dl). The normal recovery after toxin administration was not significant (40.62 ± 1.78 mg/dl).

### Effect on the end products of lipid peroxidation

**Figure 3:**
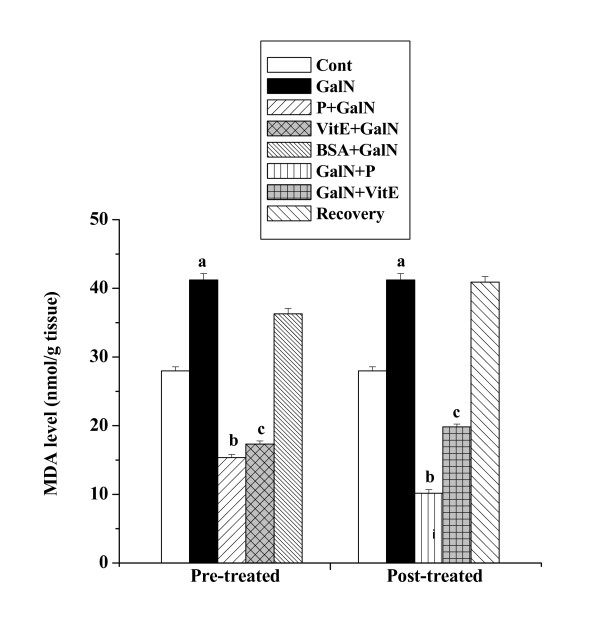
Effect of the protein on TBARS formation in GalN induced kidney damage in mice. Left and right panel show preventive and curative effect of the protein respectively. Cont: MDA content in normal mice, GalN: MDA content in only GalN treated mice, Protein + GalN: MDA level in which the protein was given prior to GalN administration, VitE + GalN: MDA level in which vitamin E was administered prior to GalN treatment, BSA + GalN: MDA level in which BSA was given before GalN administration. GalN + Protein: MDA content in which the protein was given after GalN administration, GalN + Vit E: MDA level in which vitamin E was administered post to GalN treatment and Recovery: MDA content in mice which were treated with GalN for 3 days and kept next 4 days without any treatment. Each column represents mean ± SD, n = 6. "a" indicates the significant difference between the vehicle control and toxin treated groups, "b" indicates the significant difference between the toxin treated and protein treated groups and "c" indicates the significant difference between the toxin treated and vitamin E treated groups. (P^a ^< 0.01, P^b ^< 0.01, P^c ^< 0.01).

Figure 3 shows the levels of MDA in kidney tissue homogenates obtained from all the experimental mice. MDA level (41.22 ± 0.92 nmol/g tissue) in kidney tissue homogenates of GalN treated mice was found to be higher than that level compared to normal control mice (27.99 ± 0.59 nmol/g tissue). Pre-treatment with the protein followed by toxin administration decreased the level (15.35 ± 0.45 nmol/g tissue). Post treatment with the protein after toxin administration decreased MDA level (10.17 ± 0.52 nmol/g tissue) also. The normal recovery after toxin treatment was very slow (40.91 ± 0.82 nmol/g tissue).

### Effect on antioxidant enzymes

#### SOD activities

**Figure 4:**
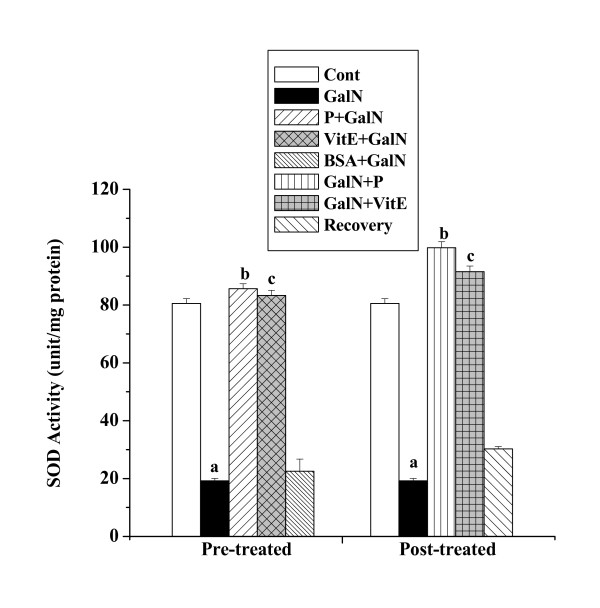
Effect of the protein on SOD activity in GalN induced kidney damage in mice. Left and right panel show preventive and curative effect of the protein respectively. Cont: SOD activity in normal mice, GalN: SOD activity in only GalN treated mice, Protein + GalN: SOD activity in which the protein was given prior to GalN administration, VitE + GalN: SOD activity in which vitamin E was administered prior to GalN treatment, BSA + GalN: SOD activity in which BSA was given before GalN administration. GalN + Protein: SOD activity in which the protein was given after GalN administration, GalN + Vit E: SOD activity in which vitamin E was administered post to GalN treatment and Recovery: SOD activity in mice which were treated with GalN for 3 days and kept next 4 days without any treatment. Each column represents mean ± SD, n = 6. "a" indicates the significant difference between the vehicle control and toxin treated groups, "b" indicates the significant difference between the toxin treated and protein treated groups and "c" indicates the significant difference between the toxin treated and vitamin E treated groups. (P^a ^< 0.01, P^b ^< 0.01, P^c ^< 0.01).

SOD activities in kidney tissue homogenates of all experimental mice are shown in figure 4. SOD activity was reduced in toxin control mice (19.21 ± 0.85 unit/mg protein) compared to the normal control (80.55 ± 1.61 unit/mg protein). There was increase in SOD activity in the protein treated mice (85.65 ± 1.71 unit/mg protein) prior to GalN administration and in protein treated mice (99.82 ± 2.09 unit/mg protein) after GalN administration. No significant normal recovery (30.25 ± 0.83 unit/mg protein) after toxin treatment was observed.

#### CAT activities

**Figure 5:**
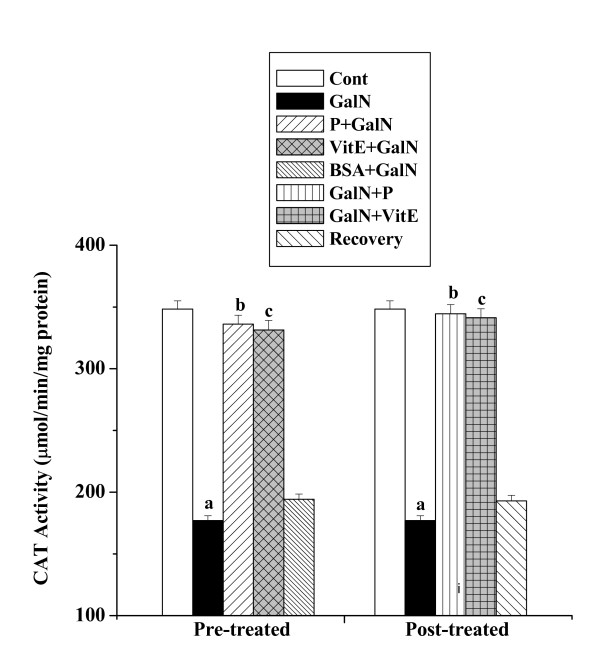
Effect of the protein on CAT activity in GalN induced kidney damage in mice. Left and right panel show preventive and curative effect of the protein respectively. Cont: CAT activity in normal mice, GalN: CAT activity in only GalN treated mice, Protein + GalN: CAT activity in which the protein was given prior to GalN administration, VitE + GalN: CAT activity in which vitamin E was administered prior to GalN treatment, BSA + GalN: CAT activity in which BSA was given before GalN administration. GalN + Protein: CAT activity in which the protein was given after GalN administration, GalN + Vit E: CAT activity in which vitamin E was administered post to GalN treatment and Recovery: CAT activity in mice which were treated with GalN for 3 days and kept next 4 days without any treatment. Each column represents mean ± SD, n = 6. "a" indicates the significant difference between the vehicle control and toxin treated groups, "b" indicates the significant difference between the toxin treated and protein treated groups and "c" indicates the significant difference between the toxin treated and vitamin E treated groups. (P^a ^< 0.01, P^b ^< 0.01, P^c ^< 0.01).

CAT activities in kidney tissue homogenates of all experimental mice are shown in figure 5. There was decreased CAT activity in toxin control (176.96 ± 3.89 μmol/min/mg protein) compared to the normal control (348.31 ± 6.62 μmol/min/mg protein). Pre as well as post-treatment with the protein increased CAT activity. The activities were 336.06 ± 7.22 μmol/min/mg protein and 344.49 ± 7.41 μmol/min/mg protein respectively. The normal recovery after toxin treatment was not significant (192.95 ± 4.51 μmol/min/mg protein).

#### GST activities

**Figure 6:**
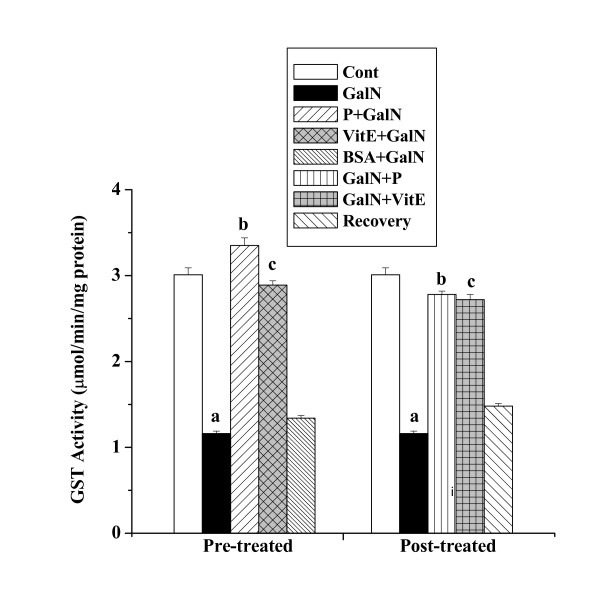
Effect of the protein on GST activity in GalN induced kidney damage in mice. Left and right panel show preventive and curative effect of the protein respectively. Cont: GST activity in normal mice, GalN: GST activity in only GalN treated mice, Protein + GalN: GST activity in which the protein was given prior to GalN administration, VitE + GalN: GST activity in which vitamin E was administered prior to GalN treatment, BSA + GalN: GST activity in which BSA was given before GalN administration. GalN + Protein: GST activity in which the protein was given after GalN administration, GalN + Vit E: GST activity in which vitamin E was administered post to GalN treatment and Recovery: GST activity in mice which were treated with GalN for 3 days and kept next 4 days without any treatment. Each column represents mean ± SD, n = 6. "a" indicates the significant difference between the vehicle control and toxin treated groups, "b" indicates the significant difference between the toxin treated and protein treated groups and "c" indicates the significant difference between the toxin treated and vitamin E treated groups. (P^a ^< 0.01, P^b ^< 0.01, P^c ^< 0.01).

GST activities in kidney tissue homogenates of all experimental mice are shown in figure 6. A decreased GST activity was observed in toxin control (1.16 ± 0.03 μmol/min/mg protein) compared to the normal control (3.01 ± 0.08 μmol/min/mg protein). Protein treatment prior to GalN intoxication caused an elevation in GST activity (3.35 ± 0.09 μmol/min/mg protein). GST activity (2.78 ± 0.04 μmol/min/mg protein) was also elevated in mice group treated with protein after toxin administration. The normal recovery after toxin treatment was very slow (1.48 ± 0.03 μmol/min/mg protein).

#### GR activities

**Figure 7:**
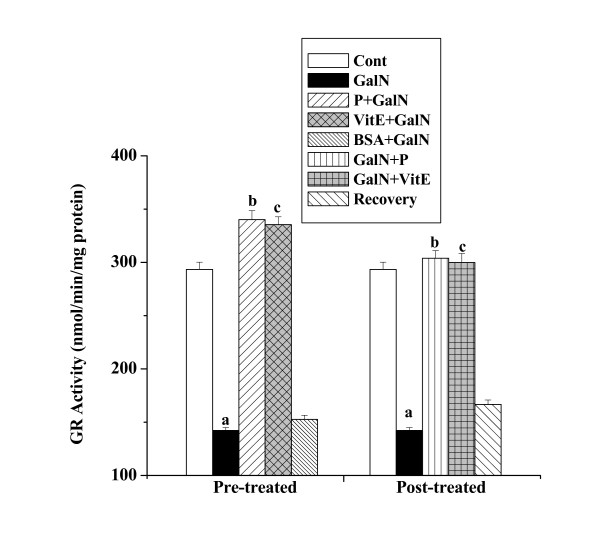
Effect of the protein on GR activity in GalN induced kidney damage in mice. Left and right panel show preventive and curative effect of the protein respectively. Cont: GR activity in normal mice, GalN: GR activity in only GalN treated mice, Protein + GalN: GR activity in which the protein was given prior to GalN administration, VitE + GalN: GR activity in which vitamin E was administered prior to GalN treatment, BSA + GalN: GR activity in which BSA was given before GalN administration. GalN + Protein: GR activity in which the protein was given after GalN administration, GalN + Vit E: GR activity in which vitamin E was administered post to GalN treatment and Recovery: GR activity in mice which were treated with GalN for 3 days and kept next 4 days without any treatment. Each column represents mean ± SD, n = 6. "a" indicates the significant difference between the vehicle control and toxin treated groups, "b" indicates the significant difference between the toxin treated and protein treated groups and "c" indicates the significant difference between the toxin treated and vitamin E treated groups. (P^a ^< 0.01, P^b ^< 0.01, P^c ^< 0.01).

GR activities in kidney tissue homogenates obtained from all the experimental mice are shown in figure 7. There was reduction of GR activity in toxin control (142.18 ± 2.74 nmol/min/mg protein) compared to the normal control (293.38 ± 6.78 nmol/min/mg protein). Elevation of GR activity (340.12 ± 8.33 nmol/min/mg protein) was observed in protein pre-treatment group. Post treatment with the protein increased GR activity (303.82 ± 7.17 nmol/min/mg protein) also. A little normal recovery (166.72 ± 4.05 nmol/min/mg protein) after toxin treatment was observed.

### Effect on cellular metabolites

#### GSH levels

**Figure 8:**
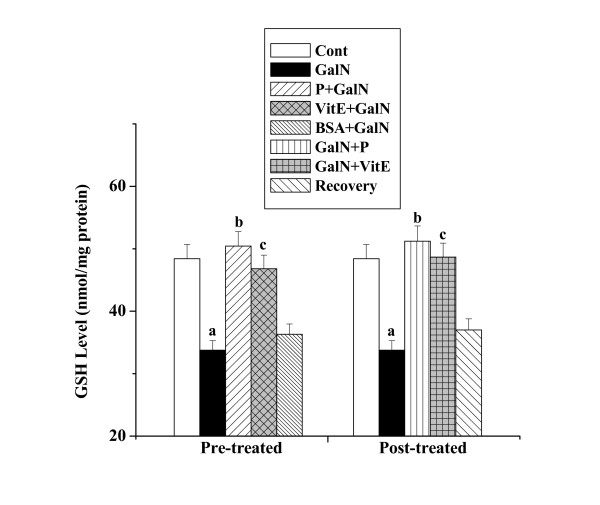
Effect of the protein on GSH level in GalN induced kidney damage in mice. Left and right panel show preventive and curative effect of the protein respectively. Cont: GSH level in normal mice, GalN: GSH level in only GalN treated mice, Protein + GalN: GSH level in which the protein was given prior to GalN administration, VitE + GalN: GSH level in which vitamin E was administered prior to GalN treatment, BSA + GalN: GSH level in which BSA was given before GalN administration. GalN + Protein: GSH level in which the protein was given after GalN administration, GalN + Vit E: GSH level in which vitamin E was administered post to GalN treatment and Recovery: GSH level in mice which were treated with GalN for 3 days and kept next 4 days without any treatment. Each column represents mean ± SD, n = 6. "a" indicates the significant difference between the vehicle control and toxin treated groups, "b" indicates the significant difference between the toxin treated and protein treated groups and "c" indicates the significant difference between the toxin treated and vitamin E treated groups. (P^a ^< 0.01, P^b ^< 0.01, P^c ^< 0.01).

Figure 8 shows the levels of GSH in kidney tissue homogenates obtained from all the experimental mice. There was decrease of GSH level in toxin control (33.77 ± 1.52 nmol/mg protein) compared to that of normal control (48.41 ± 2.28 nmol/mg protein), whereas protein pre-treatment increased the level (50.44 ± 2.32 nmol/mg protein). An increased GSH level (51.23 ± 2.43 nmol/mg protein) was observed in kidney homogenates obtained from the mice treated with the protein after toxin administration. There was no significant normal recovery (37.01 ± 1.77 nmol/mg protein) after toxin treatment.

#### GSSG levels

**Figure 9:**
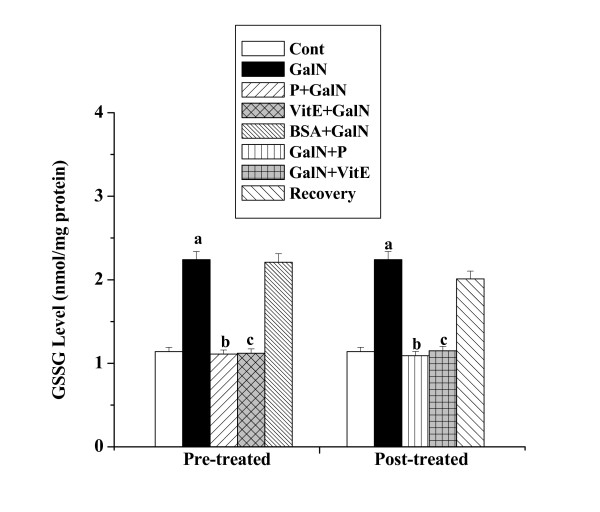
Effect of the protein on GSSG level in GalN induced kidney damage in mice. Left and right panel show preventive and curative effect of the protein respectively. Cont: GSSG level in normal mice, GalN: GSSG level in only GalN treated mice, Protein + GalN: GSSG level in which the protein was given prior to GalN administration, VitE + GalN: GSSG level in which vitamin E was administered prior to GalN treatment, BSA + GalN: GSSG level in which BSA was given before GalN administration. GalN + Protein: GSSG level in which the protein was given after GalN administration, GalN + Vit E: GSSG level in which vitamin E was administered post to GalN treatment and Recovery: GSSG level in mice which were treated with GalN for 3 days and kept next 4 days without any treatment. Each column represents mean ± SD, n = 6. "a" indicates the significant difference between the vehicle control and toxin treated groups, "b" indicates the significant difference between the toxin treated and protein treated groups and "c" indicates the significant difference between the toxin treated and vitamin E treated groups. (P^a ^< 0.01, P^b ^< 0.01, P^c ^< 0.01).

The levels of GSSG in kidney tissue homogenates obtained from all the experimental mice are shown in figure 9. GSSG level in toxin control (2.24 ± 0.098 nmol/mg protein) was found to be higher than that level compared to normal control (1.14 ± 0.051 nmol/mg protein). Pre-treatment with the protein followed by toxin administration decreased the level (1.11 ± 0.049 nmol/mg protein). GSSG level (1.09 ± 0.052 nmol/mg protein) has been found to decrease in post-treatment group of mice. The normal recovery after toxin treatment was very slow (2.01 ± 0.095 nmol/mg protein).

#### Levels of total thiols

**Figure 10:**
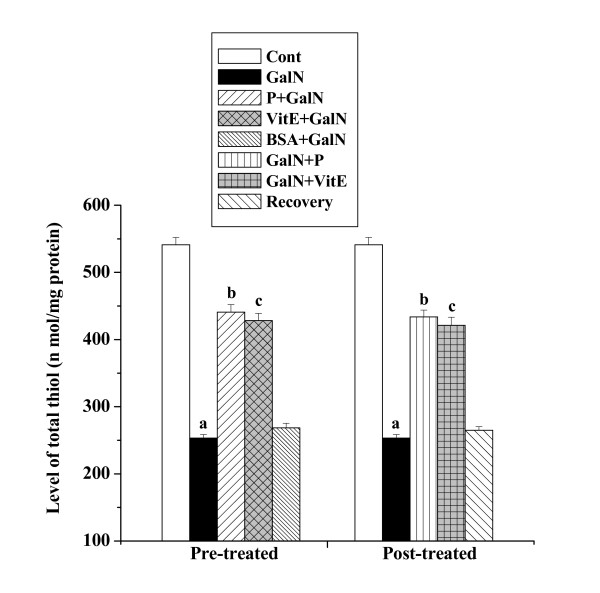
Effect of the protein on levels of total thiols in GalN induced kidney damage in mice. Left and right panel show preventive and curative effect of the protein respectively. Cont: total thiol level in normal mice, GalN: total thiol level in only GalN treated mice, Protein + GalN: total thiol level in which the protein was given prior to GalN administration, VitE + GalN: total thiol level in which vitamin E was administered prior to GalN treatment, BSA + GalN: total thiol level in which BSA was given before GalN administration. GalN + Protein: total thiol level in which the protein was given after GalN administration, GalN + Vit E: total thiol level in which vitamin E was administered post to GalN treatment and Recovery: total thiol level in mice which were treated with GalN for 3 days and kept next 4 days without any treatment. Each column represents mean ± SD, n = 6. "a" indicates the significant difference between the vehicle control and toxin treated groups, "b" indicates the significant difference between the toxin treated and protein treated groups and "c" indicates the significant difference between the toxin treated and vitamin E treated groups. (P^a ^< 0.01, P^b ^< 0.01, P^c ^< 0.01).

Figure 10 shows the levels of total thiols in kidney tissue homogenates obtained from all the experimental mice groups. Toxin treatment decreased the level of total thiols (253.28 ± 5.32 nmol/mg protein) compared to the normal control group (541.25 ± 10.83 nmol/mg protein) whereas protein pre-treatment increased the level (440.83 ± 11.25 nmol/mg protein). Post treatment of the protein after GalN intoxication increased the level of total thiols (433.73 ± 10.11 nmol/mg protein) compared to toxin control group. No significant normal recovery (264.88 ± 5.29 nmol/mg protein) after toxin treatment was found.

Vitamin E treatment pre and post to GalN intoxication could protect kidney by preventing alterations in all the antioxidant indices described in the present study, whereas BSA did not show any protective activity.

## Discussion

The aim of the present study was to investigate whether the protein isolated from the herb *Cajanus indicus *possesses any preventive as well as curative role against GalN induced renal damages. Results suggest that administration of GalN at a dose of 800 mg/kg body weight for 3 days significantly altered the status of the antioxidant defense system in the kidney. Protein treatment both pre and post to the toxin administration restored the activities of all the intracellular antioxidant enzymes as well as level of cellular metabolites.

Reports on the mechanism of GalN induced hepatotoxicity suggests that GalN inhibits the synthesis of RNA as well as protein via reduction of cellular UTP, a substrate for RNA polymerase. GalN shifts the equilibrium between the uridine phosphate consuming and producing reactions, in favor of the former via the rapid formation and accumulation of UDP amino sugars [2]. Studies from Quintero et al [47] showed that free radical dependent pathway is involved in GalN induced hepatic apoptosis and necrosis in rat hepatocytes. Mangeney-Andreani et al [10] suggested that energy metabolism of hepatocytes was also inhibited by GalN application. In addition, report from Sire et al [48] reveals that GalN damaged the enzymes involved in the transport of substrates to the mitochondria and consequently modified the phospholipid composition of the membrane. It has been observed that patient suffering with liver disease have a decrease in renal blood flow, indicating renal vasoconstriction and this effect is more prominent in those people affected with HRS [49]. Earlier studies reported that endothelin-1 (ET-1), one of the most potent vasoconstrictor in biological system, induces renal vasoconstriction as well as reduction in glomerular filtration rate (GFR), renal blood flow and sodium excretion [50]. Uchida et al [51] and Sakamoto et al [52] reported that various cells within the kidney tissue either express mRNA for ET-1 or can synthesis ET-1 and thus it reveals that ET-1 may, therefore, have a paracrine or autocrine effect. It has been observed that the levels of plasma ET-1 concentration are elevated in patients with severe liver disease and HRS [53]. Annand et al [8] reported that GalN intoxication causes liver injury followed by renal impairment, which is manifested by the decrease in renal blood flow, creatinine clearance as well as increase in plasma ET-1 concentration and also up-regulation of ET-1 receptor in renal cortex although kidneys are histologically normal. Thus study also suggests that renal failure also appears to be secondary to liver failure.

In the present study we found that GalN administration at a dose of 800 mg/kg body weight for three days caused enhanced oxidative insult in the kidney tissue as evidenced by the increased renal TBARS level and depletion in the activities of endogenous antioxidant enzymes such as SOD, CAT, GR and GST. GalN intoxication also reduced the level of total thiol and GSH followed by the increase in GSSG level. The renal dysfunction due to GalN treatment was also manifested by the increase in serum creatinine and UN levels as compared to the normal group of mice. Protein treatment at a dose of 2 mg/kg body weight for 4 days both prior and post to the toxin administration attenuated the increased creatinine and UN levels compared to the toxin control. Administration of the protein restored the activities of intracellular antioxidant enzymes SOD, CAT, GR and GST. The decreased level of ROS scavengers like GSH and total thiols were also enhanced by the protein treatment both prior and post to the GalN intoxication. The content of total thiols includes the protein-bound thiols as well as nonprotein-bound thiols. It has been reported that the former usually contribute more (a few fold higher than the later) to the total thiols contents [54]. The protein also ameliorated the enhanced level of TBARS as well as the level of GSSG compared to the toxin control.

As reported from the earlier studies of our laboratory, the protein could scavenge DPPH radicals as well as restore the activities of different antioxidant enzymes and attenuate various toxin-induced enhancement of lipid peroxidation [31-34]. The detail mechanism of action of this protein necessary for the protective action against hepatic and renal damages is not yet fully known. As it possesses hepatoprotective activity, it appears that the amelioration of renal damage may not be its direct action to the kidney. GalN induced renal failure appears at the end stage of liver cirrhosis; so protecting the liver from that severe damage it indirectly possesses protective role against GalN-induced disorders in the kidney.

Although a number of very recent reports revealed that protein molecules from various plant sources possess antioxidant and hepatoprotective activities like the protein of our interest [55-57], similarities among their structural features could not be related at this stage because of the lack of knowledge on their structures. These reports also lack the knowledge of the exact mechanism of actions of those protein molecules; only some biochemical properties are known and that could be compared to the protein of our interest.

## Conclusion

In conclusion we would like to mention that GalN administration caused oxidative insult in the kidney. Protein treatment both prior and post to the toxin administration normalize that stress in the organ. As GalN induced renal failure seems to occur at the end stage of liver cirrhosis, the protective role of the protein against GalN induced renal damages is likely to be an indirect effect probably comes to play via the protection of hepatic disorders. Further works are however needed to define the exact mechanisms by which the protein exhibits this protective action and are currently in progress.

## Competing interests

The author(s) declare that they have no competing interests.

## Authors' contributions

MS: made substantial contributions to the design of the study, the collection of the data as well as the interpretation and analysis of the data and drafted the manuscript.

PM: made substantial contributions to the design of the study, the collection of the data as well as the interpretation and analysis of the data and drafted the manuscript.

PCS: the investigation-in-charge for the study, guided MS and PM for all the experiments, interpretation and analysis of the data as well as edited the manuscript.

All authors read and approved the final manuscript.

## Pre-publication history

The pre-publication history for this paper can be accessed here:


